# Event extraction for DNA methylation

**DOI:** 10.1186/2041-1480-2-S5-S2

**Published:** 2011-10-06

**Authors:** Tomoko Ohta, Sampo Pyysalo, Makoto Miwa, Jun’ichi Tsujii

**Affiliations:** 1 Department of Computer Science, University of Tokyo, Tokyo, Japan; 2 School of Computer Science, University of Manchester, Manchester, UK; 3 National Centre for Text Mining, University of Manchester, Manchester, UK

## Abstract

**Background:**

We consider the task of automatically extracting DNA methylation events from the biomedical domain literature. DNA methylation is a key mechanism of epigenetic control of gene expression and implicated in many cancers, but there has been little study of automatic information extraction for DNA methylation.

**Results:**

We present an annotation scheme for DNA methylation following the representation of the BioNLP shared task on event extraction, select a set of 200 abstracts including a representative sample of all PubMed citations relevant to DNA methylation, and introduce manual annotation for this corpus marking nearly 3000 gene/protein mentions and 1500 DNA methylation and demethylation events. We retrain a state-of-the-art event extraction system on the corpus and find that automatic extraction of DNA methylation events, the methylated genes, and their methylation sites can be performed at 78% precision and 76% recall.

**Conclusions:**

Our results demonstrate that reliable extraction methods for DNA methylation events can be created through corpus annotation and straightforward retraining of a general event extraction system. The introduced resources are freely available for use in research from the GENIA project homepage http://www-tsujii.is.s.u-tokyo.ac.jp/GENIA.

## Background

During the previous decade of concentrated study of biomedical information extraction (IE), most efforts have focused on the foundational task of detecting mentions of entities of interest and the extraction of simple associations between these entities, typically represented as binary relations [1-3]. However, in recent years there has been increased interest in biomolecular event extraction using representations that capture typed, structured *n*-ary associations of entities in specific roles, such as *regulation* of the *phosphorylation* of a specific *domain* of a particular *protein*[4-7]. The state of the art in such extraction methods was evaluated in the BioNLP’09 Shared Task on Event Extraction (below, BioNLP ST) [8], and event extraction following the BioNLP ST model has continued to draw interest also after the 2009 task, with recent work including advances in extraction methods [9-12], the release of extraction system software and large-scale automatically annotated data [13,14] and the development of additional annotated resources following the event representation [15,16] as well as a follow-up shared task in 2011 [17,18]. Of the findings of the BioNLP ST evaluation, it is of particular interest to us that the highest-performing methods include many that are purely machine-learning based [8], learning what to extract directly from a corpus annotated with examples of the events of interest. This implies that state-of-the-art extraction methods for new types of events can be created by providing annotated resources to an existing system, without the need for direct development of natural language processing or IE methods. We recently applied such an annotation-based approach to the automatic extraction of five types of protein post-translational modification events [15]. While this study demonstrated the feasibility of the approach, extraction performance was somewhat low, with analysis indicating training data size as a limiting factor. Here, we apply a similar approach to DNA methylation, a specific and biologically highly relevant event type not considered in previous event extraction studies. Focus on a single event type was expected to allow more reliable extraction through increased training data and analysis of the requirements for training accurate extraction methods.

In the following, we first outline the biological significance of DNA methylation and discuss existing resources. We then introduce the event extraction approach applied, describe the new annotated corpus created in this study, and present event extraction results using a method trained on the corpus.

### DNA methylation

The term *epigenetics* refers to the study of molecular mechanisms “beyond genetics” that cause inheritable changes of gene expression and/or phenotype without alteration of the DNA sequence. Such mechanisms are today understood to play an important role in many biological processes, including the genetic program for development, cell differentiation, and tissue-specific gene expression. DNA methylation was first suggested as an epigenetic mechanism for the control of gene activity during development in 1975 [19,20], and the role of DNA methylation in cancer was first reported in 1987 [21]. DNA methylation of CpG islands in gene promoter regions is now understood to be one of the most consistent genetic alterations in cancer, and DNA methylation is a prominent area of study.

Chemically, DNA methylation is a simple reaction adding a methyl group to a specific position of a cytosine pyrimidine ring or an adenine purine ring. While a single nucleotide can only be either methylated or unmethylated, in text the overall degree of promoter methylation is often reported as *hypo-* or *hyper-methylation*, with hyper-methylation implying that the expression of a gene is silenced. Because of the precise definition of the phenomenon and the relatively specific terms in which it is typically discussed in publications, we expected it to provide a well-defined target for annotation and automatic extraction.

### DNA methylation in PubMed

**Figure 1:**
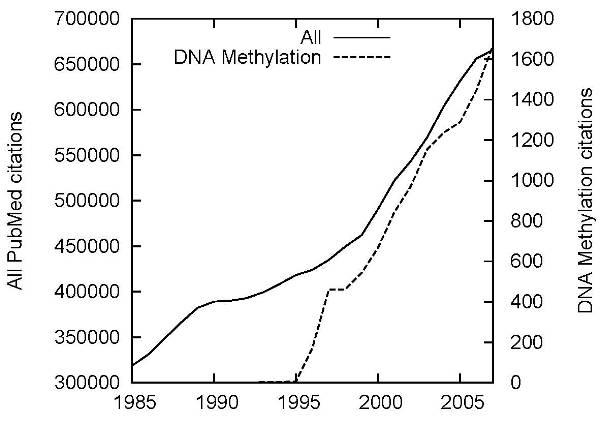
**DNA methylation in PubMed**. Citations tagged with the MeSH term *DNA Methylation* compared to all citations in PubMed by publication year. Note different scales.

We follow common practice in biomedical IE in drawing texts for our corpus from PubMed abstracts. Currently containing more than 20 million citations for biomedical literature (over 11M with abstracts) and growing exponentially [22], the literature database provides a rich resource for IE and text mining. To facilitate access to documents relevant to specific topics, each PubMed citation is manually assigned terms that identify its primary topics using MeSH, a controlled vocabulary of over 25,000 terms. MeSH contains also a *DNA Methylation* term, allowing specific searches for citations on the topic. Figure 1 shows the number of citations per year of publication matching this term contrasted with overall citations, illustrating explosive growth of interest in DNA methylation, outstripping the overall growth of the literature. Particular increases can be seen after the introduction of DNA microarrays for monitoring gene expression [23] and the introduction of high-throughput screening methods [24,25]. The total number of PubMed citations tagged with the *DNA Methylation* MeSH term at the time of this writing is 16734 (15557 of which have an abstract). The large number of documents tagged as relating to this topic and the human judgments assuring their relevance make querying for this term a natural choice for selecting texts for annotation. However, direct PubMed query as the only selection strategy would ignore significant existing resources, discussed in the following.

### DNA methylation databases

A growing number of databases collating information on DNA methylation are becoming available. The first such database, MethDB [26], was introduced in 2001 and remains actively developed. MethDB contains PubMed citation references as evidence for contained entries, but no more specific identification of the expressions stating DNA methylation events. The methPrimerDB [27] database provides additional information on PCR primers on top of MethDB, but does not add further specification of the methylated gene or text-bound annotation. PubMeth [28] is a database of DNA methylation in cancer with evidence sentences from the literature, initially selected by PubMed query for “more than 15 methylation-related keywords” (e.g. *DNA methylation*, *methylated*, *epigenetic*). This database stores information on cancer types and subtypes, methylated genes and the experimental method used to identify methylation, as well as annotated evidence sentences. MeInfoText [29] is a database of DNA methylation and cancer information automatically extracted from PubMed documents matching the query terms *human*, *methylation and cancer* using term co-occurrence statistics. Of the DNA Methylation resources, only PubMeth and MeInfoText contain text-bound annotation identifying specific spans of characters containing the gene mention and stating the DNA methylation. In this study, we consider specifically PubMeth as a source of reference text-bound annotations due to availability and the ability to redistribute derived data. Initial text-bound annotations in PubMeth were generated using keyword lookup, but the database annotations are manually reviewed. Table 1 shows example evidence sentences from PubMeth and their annotated spans. While the PubMeth annotation differs from the BioNLP ST representation in a number of ways, such as not separating coordinated entities (Table 1c) and not annotating methylation sites (Table 1d), it provides both a reference identifying annotation targets from a biologically motivated perspective and a potential starting point for full event annotation.

**Table 1:** Examples of PubMeth evidence sentence annotation

a)	MS-PCR revealed the [methylation] of the [*p16*] gene in 10 (34%) of 29 [**NSCLCs**]
b)	30% (27 of 91) of [**lung tumors**] showed [hypermethylation] of the 5’CpG region of the [*p14ARF gene*]
c)	[Promotor hypermethylations] were detected in [*O6-methylguanine-DNA methyltransferase* (*MGMT*), *RB1*, *estrogen receptor*, *p73*, *p16INK4a*, *death-associated protein kinase*, *p15INK4b*, *and p14ARF*]
d)	The promoter region of the [*p16INK4*] gene was [hypermethylated] in the tumor samples of the primary or metastatic site

Annotated spans delimited by brackets and statements expressing methylation underlined, gene mentions shown in italics, and cancer mentions in bold.

### Annotation

For annotation, we adapted the representation applied in the BioNLP ST on event extraction with minimal changes in order to allow systems developed for the task to be applied also for the newly annotated corpus. Documents were selected following the basic motivation presented above, with reference to the requirements specified by the annotation scheme, and some automatic preprocessing was applied as annotator support. This section details the annotation approach.

### Entity and event representation

For the core named entity annotation, we primarily follow the gene/gene product (GGP) annotation criteria applied for the BioNLP ST data [30]. In brief, the guidelines specify annotation of minimal contiguous spans containing mentions of specific gene or gene product (RNA/protein) names, where *specific name* is understood to be one that would allow a biologist to identify the corresponding entry in a gene/protein database such as Uniprot or Entrez Gene. The annotation thus excludes, for example, names of gene/protein families and complexes. A single annotation type, *Gene or gene product*, is applied without distinction between genes and their products, and normalization of the tagged strings to gene/protein database entries is not performed as part of the annotation effort.

These strict guidelines were followed in the annotation of previously unannotated documents, but for compatibility with PubMeth annotations we relaxed the specificity requirement in the reannotation of documents included in the database, allowing the annotation of, for example, gene or gene product families when these were annotated in PubMeth.

In addition to the identification of the modified gene, to fully characterize a DNA methylation event it is important to identify the site of the modification. We marked mentions of sites as *DNA domain or region* terms following the original GENIA term corpus annotation guidelines [31]. As in the BioNLP ST data, site mentions were only marked when the sites are relevant to one or more events. Thus, unlike the GGP and event annotations, the *DNA domain or region* annotations are not exhaustive.

**Figure 2:**
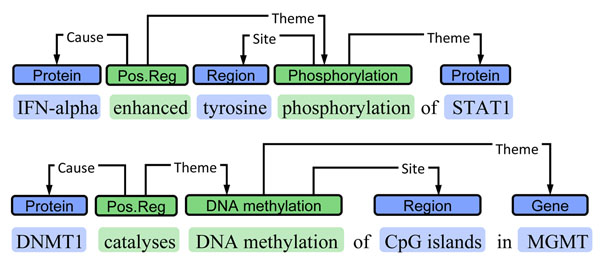
**Event representation**. BioNLP Shared Task representation for annotation of phosphorylation events (above) and representation applied for DNA methylation (below).

For representing DNA methylation events, the annotation applied to capture protein phosphorylation events in the BioNLP ST task 2 closely matched the needs for DNA methylation (Figure 2). While the Site arguments of the ST *Phosphorylation* events are protein domains, machine-learning based extraction methods should be able to associate this role with DNA domains given training data. We thus adopted a representation where *DNA methylation* events are associated with a gene/gene product as their Theme and a *DNA domain or region* as Site. Each event is also associated with a particular span of text expressing it, termed the *event trigger*. Annotators were instructed to always mark some trigger expression, using the best approximation in cases where no unambiguous trigger (e.g. *methylates*) was present. We note that while we do not here specifically distinguish degrees of methylation (e.g. *methylation* from *hyper-methylation*), the trigger annotations are expected to facilitate adding these distinctions if necessary for particular applications: statements identifying the degree of methylation are likely to be found in the close context of the expression stating the methylation event. We further initially marked catalysts using *Positive regulation* events following the BioNLP ST model, but dropped this class of annotation as a sufficient number of examples was not found in the corpus.

The event types of the BioNLP ST are drawn from the GENIA Event ontology [5], which in turn draws its type definitions from the community-standard Gene Ontology (GO) [32]. To maintain compatibility with these resources, we opted to follow the GO also for the definition of the new event type considered here. GO defines DNA methylation as:

The covalent transfer of a methyl group to either N-6 of adenine or C-5 or N-4 of cytosine.

We note that while the definition may appear restrictive, methylation of adenine N-6 or cytosine C-5/N-4 encompasses the entire set of ways in which DNA can be methylated. To GO definition could thus be adopted without limitation to the scope of the annotation.

### Document selection

The selection of source documents for an annotated corpus is critical for assuring that the corpus provides relevant and representative material for studying the phenomena of interest. Domain corpora frequently consist of documents from a particular subdomain of interest: for example, the GENIA corpus focuses on documents concerning transcription factors in human blood cells [31]. Methods trained and evaluated on such focused resources will not necessarily generalize well to broader domains. However, there has been little study of the effect of document selection on event extraction performance. Here, we applied two distinct strategies to get a representative sample of the full scope of DNA methylation events in the literature and to assure that our annotations are relevant to the interests of biologists and our results applicable to the overall distribution of DNA methylation events in the literature.

In the first strategy, we aimed in particular to select a representative sample of documents relevant to the targeted event types. For this purpose, we directly searched the PubMed literature database. We further decided not to include any text-based query in the search to avoid biasing the selection toward particular entities or forms of event expression. Instead, we only queried for the single MeSH term *DNA Methylation*. This term has the PubMed annotation scope definition:

Addition of methyl groups to DNA. DNA methyltransferases (DNA methylases) perform this reaction using S-ADENOSYLMETHIONINE as the methyl group donor.

While this definition of DNA Methylation takes a different perspective than the GO definition adopted for the event specification, in practice it identifies the same concept: by definition, DNA methylation is only performed by DNA methyltranferases, and the mentioned donor is the only one presently known. We can thus expect that PubMed queries for this concept match a complete and unbiased set of documents involving the targeted concepts.

**Figure 3:**
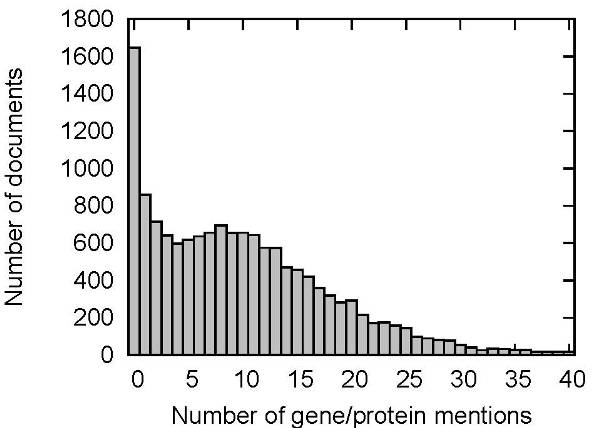
**Gene/protein mentions in DNA methylation abstracts**. Number of abstracts with given number of automatically tagged gene/protein mentions.

While search for documents that are indexed by humans with the MeSH term *DNA Methylation* is expected to provide high-precision results for the full topic, not all such documents necessarily discuss events where specific genes are methylated. In initial efforts to annotate a random sample of these documents, we found that many did not mention specific gene names. To reduce wasted effort in examining documents that contain no markable events, we added a filter requiring a minimum number of (likely) gene mentions. We first tagged all citations tagged with *DNA Methylation* that have an abstract in PubMed (14350 at the time of selection) using the BANNER protein/gene name tagger [33] trained on the GENETAG corpus [34]. We found that while the overwhelmingly most frequent number of tagged mentions per document is zero, a substantial mass of abstracts have large mention counts (Figure 3). We note that as the tagger has been evaluated at 86% F-score on a broad-coverage corpus [34], it is unlikely to severely misestimate the true distribution. We decided after brief preliminary experiments to filter the initial selection of documents to include only those in which at least 5 gene/protein mentions were marked by the automatic tagger. This excludes most documents without markable events without introducing obvious other biases.

In the second strategy, we extended and completed the annotation of a random selection of PubMeth evidence sentences, aiming to leverage existing resources and to select documents that had been previously judged relevant to the interests of biologists studying the topic. This provides an external definition of document relevance and allows us to estimate to what extent the applied annotation strategy can capture biologically relevant statements. This strategy is also expected to select a concentrated, event-rich set of documents. However, the selection will also necessarily carry over biases toward particular subsets of relevant documents from the original selection [28] and will not be a representative sample of the overall distribution of such documents in the literature.

For producing the largest number of event annotations with the least effort, the most efficient way to use the PubMeth data would have been to simply extract the evidence sentences and complete the annotation for these. However, viewing the context in which event statements occur as centrally important, we opted to annotate complete abstracts, with initial annotations from PubMeth evidence sentences automatically transferred into the abstracts. We note that not all PubMeth evidence spans were drawn from abstracts, and not all that were matched a contiguous span of text. We could align PubMeth evidence annotations into 667 PubMed abstracts (approximately 57% of the referenced PMID number in PubMeth) and completed event annotation for a random sample of these.

### Document preprocessing

To reduce annotation effort, we applied automatic systems to produce initial candidate sentence boundaries and GGP annotations for the corpus. For sentence splitting, we applied the GENIA sentence splitter [35], and for gene/protein tagging, we applied the BANNER NER system [33] trained on GENETAG [34] (as for document filtering). The GENETAG guidelines and gene/protein entity annotation coverage are known to differ from those applied for GGP annotation here [36]. However, the broad coverage of PubMed provided by the GENETAG suggests taggers trained on the corpus are likely to generalize to new subdomains such as that considered here. By contrast, all annotations that we are aware of that follow the GGP guidelines are subdomain-specific.

We note that all annotations in the produced corpus are at a minimum confirmed by a human annotator and that events are annotated without performing initial automatic tagging to assure that no bias toward particular extraction methods or approaches is introduced.

## Results

### Corpus statistics

We annotated 100 abstracts following each of the two document selection strategies. The statistics of the resulting corpus are given in Table 2. There are some notable differences between the subcorpora created using the different selection strategies. While the subcorpora are similar in size, the PubMeth GGP count is 1.4 times that of the PubMed subcorpus – perhaps affected by the PubMeth entity annotation criteria – yet roughly equal numbers of methylation sites are annotated in the two. This difference is even more pronounced in the statistics for event arguments, where two thirds of PubMeth subcorpus events contain only a Theme argument identifying the GGP, while events where both Theme and Site are identified are more frequent in the other subcorpus. (The overall number of annotated sites is less than the number of events with a Site argument as the annotation criteria only call for annotating a site entity when it is referred to from an event, and multiple events can refer to the same site entity.) As the extraction of events specifying also sites is known to be particularly challenging [8], these statistics suggest the PubMed subcorpus may represent a more difficult extraction task. Only very few DNA demethylation events are found in either subcorpus, suggesting that a separate document selection strategy is necessary to assure substantial coverage of the reverse modification type. Overall, the PubMeth subcorpus contains nearly twice as many event annotations as the PubMed one, indicating that the focused document selection strategy was successful in identifying particularly event-rich abstracts.

**Table 2:** Corpus statistics

	PubMeth	PubMed	Total
Abstracts	100	100	200
Sentences	1118	1009	2127

Entities			
GGP	1695	1195	2890
Site	240	234	474

Total	1935	1429	3364

Events			
Theme only	660	214	874
Theme and Site	323	297	620

DNA methylation	977	485	1462
DNA demethylation	6	26	38

Total	983	511	1494

### Annotation quality

The annotation was performed by three experienced annotators with a molecular biology background, with one coordinating annotator with extensive experience in domain event annotation organizing and supervising the overall process.

To measure the consistency of the produced annotation, we performed independent double annotation for 20% of the corpus abstracts. These abstracts were all selected from the PubMed subcorpus, for which annotation was created without initial human annotation as reference. As the PubMeth subcorpus annotation was created using partial human annotation as a starting point, agreement is expected to be higher on the PubMeth subcorpus than on the PubMed subcorpus. This experiment should thus provide a lower bound on the overall consistency of the corpus.

We first measured agreement on the gene/gene product (GGP) entity annotation, and found very high agreement among 935 entities marked in total by the two annotators: 91% F-score using exact match criteria and 97% F-score using the relaxed “overlap” criterion where any two overlapping annotations are considered to match. We note that the high agreement is not due to annotators simply agreeing with the automatic initial annotation: the F-score of the automatic tagger against the two sets of human annotations was 65%/66% for exact and 85%/86% for overlap match. We then separately measured agreement on event annotations for those events that involved GGPs on which the annotators agreed, using the standard criteria described in the section on Evaluation Criteria below. Agreement on event annotations was also high: 84% F-score overall (85% for DNA methylation and 75% for DNA demethylation) over a total of 442 annotated events.

The overall consistency of the annotation depends on joint annotator agreement on the GGP and event annotations. However, in experimental settings such as that of the BioNLP ST where gold GGP annotation is assumed as the starting point for event extraction, measured performance is not affected by agreement on GGPs and thus arguably only the latter factor applies. As this setting is adopted also in the present study, annotation consistency suggests a human upper bound no lower than 84% F-score on extraction performance.

Estimates of the annotation consistency of biomedical domain corpora are regrettably seldom provided, and to the best of our knowledge ours is the first published estimate of inter-annotator agreement for a corpus following the event representation of the BioNLP ST. Given the complexity of the annotation – typed associations of event trigger, theme and site – the agreement compares favorably to e.g. the reported 67% inter-annotator F-score reported for protein-protein interactions on the ITI TXM corpora [37] as well as to the full event agreement for the GREC corpus [6].

### Event extraction method

To estimate the feasibility of automatic extraction of DNA methylation events and the suitability of presently available event extraction methods to this task, we performed experiments using the EventMine event extraction system of Miwa et al. [9]. On the task 2 of the BioNLP ST dataset, the benchmark most relevant to our task setting, the applied version of EventMine was recently evaluated at 55% F-score [38], outperforming the best task 2 system in the original shared task [39] by more than 10% points. To the best of our knowledge, this system represents the state of the art for this event extraction task.

EventMine is an SVM-based machine learning system following the pipeline design of the best system in the BioNLP ST [40], extending it with refinements to the feature set, the use of a machine learning module for complex event construction, and the use of two parsers for syntactic analysis [9]: the HPSG-based deep parser Enju [41] using the high-speed parsing setting (“mogura”) and the GDep [42] native dependency parser, both with biomedical domain models based on the GENIA treebank data [43]. (We note that while EventMine is not presently publicly released, the system that its design is based on [40] is available [44], has broadly comparable performance, and allows retraining.)

For evaluation, we applied a version of the BioNLP ST evaluation tools [45] modified to recognize the novel event types.

### Evaluation criteria

We followed the basic task setup and primary evaluation criteria of the BioNLP ST. Specifically, we followed task 2 (“event enrichment”) criteria, requiring for the correct extraction of a DNA methylation or demethylation event both the identification of the modified gene (GGP entity) and the identification of the modification site (*DNA domain or region* entity) when stated. As in the shared task, human annotation for GGP entities was provided as part of the system input but other entities were not, so that the system was required to identify the mentioned modification sites.

The performance of the system was evaluated using the standard precision, recall and F-score metrics for the recovery of events, with event equality defined following the “Approximate span” matching criterion applied in the primary evaluation for the BioNLP ST. This criterion relaxes strict matching requirements so that a detected event trigger or entity is considered to match a gold trigger/entity if its span is entirely contained within the span of the gold trigger, extended by one word both to the left and to the right.

### Experimental setup

We divided the corpus into three parts, first setting one third of the abstracts aside as a held-out test set and then splitting the remaining two thirds in a roughly 1:3 ratio into a training set and a development test set, giving 100 abstracts for training, 34 for development, and 66 for final test. The splits were performed randomly, but sampling so that each set has an equal number of abstracts drawn from the PubMeth and PubMed subcorpora.

The EventMine system has a single tunable threshold parameter that controls the tradeoff between system precision and recall. We first set the tradeoff using a sparse search of the parameter space [0:1], evaluating the performance of the system by training on the training set and evaluating on the development set. As these experiments did not indicate any other parameter setting could provide significantly better performance, we chose the default threshold setting of 0.5. To study the effect of training data size on performance, we performed extraction experiments randomly downsampling the training data on the document level with testing on the development set. In final experiments EventMine was trained on the combined training and development data and performance evaluated on the held-out test data.

### Extraction performance

Table 3 shows extraction results on the held-out test data. While DNA methylation events could be extracted quite reliably, the system performed poorly for DNA demethylation events. The latter result is perhaps not surprising given their small number – only 38 in total in the corpus – and indicates that a separate selection strategy is necessary to provide resources for learning the reverse reaction. Overall performance shows a small preference for precision over recall at 77% F-score. We view this level of performance very good as a first result for the new event type.

**Table 3:** Overall extraction performance

Event type	precision	recall	F-score
DNA methylation	77.6%	77.2%	77.4%
DNA demethylation	100.0%	11.1%	20.0%

Total	77.7%	76.0%	76.8%

To evaluate the relative difficulty of the extraction tasks that the two subcorpora represent and their merits as training material, we performed tests separating the two (Table 4). As predicted from corpus statistics, the PubMed subcorpus represents the more challenging extraction task. When testing on a single subcorpus, results are, unsurprisingly, better when training data is drawn from the same subcorpus; however, training on the combined data gives the best performance for all three test sets, indicating that the subcorpora are compatible.

**Table 4:** Extraction performance by subcorpus F-score performance shown.

	Test set		
Training set	PubMed	PubMeth	Both
PubMed	64.9%	71.2%	71.6%
PubMeth	62.9%	80.0%	74.0%

Both	66.2%	82.5%	76.8%

F-score performance shown.

**Figure 4:**
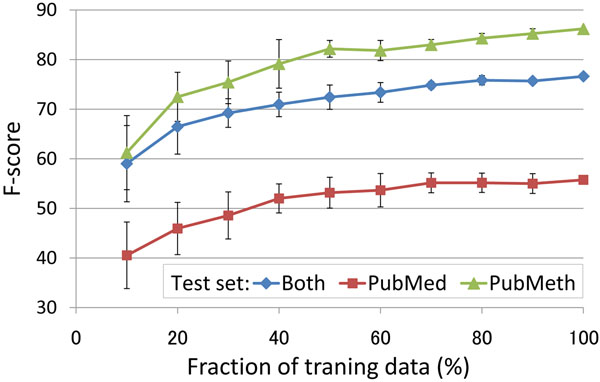
**Learning curves.** Learning curves for the two subcorpora and their combination. Both subcorpora used for training, development sets for testing. Average and error bars calculated by 10 repetitions of random subsampling of training data.

The learning curve (Figure 4) shows relatively high performance and rapid improvement for modest amounts of data, but performance improvement with additional data levels out relatively fast, nearly flattening as use of the training data approaches 100%. This suggests that extraction performance for this task is not primarily limited by training data size and that additional annotation following the same protocol is unlikely to yield notable improvement in F-score without a substantial investment of resources. As performance for the PubMed subcorpus (for which inter-annotator agreement was measured) is not yet approaching the limit implied by the corpus annotation consistency, the results suggest further need for the development of event extraction methods to improve DNA methylation event extraction.

## Related work

DNA methylation and related epigenetic mechanisms of gene expression control have been a focus of considerable recent research in biomedicine. There are many excellent reviews of this broad field; we refer the interested reader to [46,47].

There is a wealth of recent related work also on event extraction. In the BioNLP’09 shared task, 24 teams participated in the primary task and six teams in Task 2 which mostly resembles our setup in that it also required the detection of modified gene/protein and modification site. The top-performing system in Task 2 [39] achieved 44% F-score, and the highest performance reported since that we are aware of is 55% F-score for EventMine [9]. The performance we achieved for DNA methylation is considerably better than this overall result, essentially matching the best reported performance for Phosphorylation events, which we previously argued to be the closest shared task analogue to the new event category studied here. Nevertheless, direct comparison of these results may not be meaningful due to confounding factors. The only text mining efforts specifically targeting DNA methylation that we are aware of are those performed for the initial annotation of the PubMeth and MeInfoText databases [28,29], both applying approaches based on keyword matching. However, neither of these studies report results for instance-level extraction of methylation statements, and the keyword matching approaches applied in these efforts do not provide the level of detail required for evaluation against an event-annotated resource, precluding direct comparison. The present study is in many aspects similar to our previous work targeting protein post-translational modification events [15]. In this work, we annotated 422 events of 7 different types and showed that retraining an existing event extraction system allowed these to be extracted at 42% F-score. Our approach here differs from this previous work in particular in its larger scale and concentrated focus on a specific event type of high interest, reflected also in the results: while extraction performance in our previous work was limited by training data size, in the present study notably higher extraction performance was achieved and a plateau in performance with increasing data reached.

## Discussion and future work

We have presented a study of the automatic extraction of DNA methylation events from literature through annotation following the BioNLP’09 shared task event representation and the use of a retrainable event extraction system. We created a corpus of 200 publication abstracts selected to include a representative sample of DNA methylation statements from all of PubMed and manually annotated for nearly 3000 mentions of genes and gene products, 500 DNA domain or region mentions, and 1500 DNA methylation and demethylation events. Evaluation using the EventMine system showed that DNA methylation events can be extracted at 78% precision and 76% recall by retraining a previously introduced event extraction system with this corpus. The learning curve suggested that the corpus size is sufficient and that future efforts in DNA methylation event extraction should focus on extraction method development.

One natural direction for future work is to apply event extraction systems trained on the newly introduced data to abstracts available in PubMed and full texts available at PMC to create a detailed, up-to-date repository of DNA methylation events at full literature scale. Such an effort would require gene name normalization and event extraction at PubMed scale. While substantial challenges remain for accurate normalization and event extraction at this scale, both have recently been shown to be technically feasible using methods competitive with the state of the art [14,48]. Further combining the extracted events with cancer mention detection could provide a valuable resource for epigenetics research.

The newly annotated corpus, the first resource annotated for DNA methylation using the BioNLP shared task event representation, is freely available for use in research from the GENIA project homepage [49]. DNA methylation event extraction following the model developed in this study is included as part of the Epigenetics and Post-translational Modification task of the BioNLP Shared Task 2011 [17,50].

## Competing interests

The authors declare that they have no competing interests.

## Authors’ contributions

TO and SP conceived of and designed the study and drafted the manuscript. TO coordinated the annotation effort. MM performed the event extraction experiments and drafted their description. JT participated in the study design and coordination and helped to draft the manuscript. All authors read and approved the final manuscript.
